# Comparison of Different Classification Methods for Analyzing Electronic Nose Data to Characterize Sesame Oils and Blends

**DOI:** 10.3390/s151026726

**Published:** 2015-10-21

**Authors:** Xiaolong Shao, Hui Li, Nan Wang, Qiang Zhang

**Affiliations:** 1 College of Food Science and Engineering/Collaborative Innovation Center for Modern Grain Circulation, Safety/Key Laboratory of Grains and Oils Quality Control and Processing, Nanjing University of Finance and Economics, Nanjing 210023, China; E-Mails: lihui0104@foxmail.com (H.L.); wangnan0910@163.com (N.W.); 2 Department of Biosystems Engineering, University of Manitoba, Winnipeg, MB R3T 5V6, Canada; E-Mail: Qiang.Zhang@UManitoba.ca

**Keywords:** electronic nose, pressed sesame oil, linear discriminant analysis, k-nearest neighbor algorithm, partial least squares discriminant analysis, support vector machine, lasso, partial least squares regression

## Abstract

An electronic nose (e-nose) was used to characterize sesame oils processed by three different methods (hot-pressed, cold-pressed, and refined), as well as blends of the sesame oils and soybean oil. Seven classification and prediction methods, namely PCA, LDA, PLS, KNN, SVM, LASSO and RF, were used to analyze the e-nose data. The classification accuracy and MAUC were employed to evaluate the performance of these methods. The results indicated that sesame oils processed with different methods resulted in different sensor responses, with cold-pressed sesame oil producing the strongest sensor signals, followed by the hot-pressed sesame oil. The blends of pressed sesame oils with refined sesame oil were more difficult to be distinguished than the blends of pressed sesame oils and refined soybean oil. LDA, KNN, and SVM outperformed the other classification methods in distinguishing sesame oil blends. KNN, LASSO, PLS, and SVM (with linear kernel), and RF models could adequately predict the adulteration level (% of added soybean oil) in the sesame oil blends. Among the prediction models, KNN with k = 1 and 2 yielded the best prediction results.

## 1. Introduction

Pressed sesame oils are very popular in Asia [1]. Pressed sesame oil is produced by either cold pressing or hot pressing of sesame seeds. Cold pressing is normally conducted at the room temperature, and hot pressing at high temperature (180–200 °C). Before pressing, sesame seeds may be roasted and dehulled, as a pretreatment. Pressed oil may also be refined after pressing to further improve its appearance and aroma. Different pressing methods, coupled with different pretreatments and refining, result in distinct characteristics of sesame oil in terms of its physical appearance (e.g., color), chemical constituents (e.g., vitamins and sesamol), and aroma [2,3]. There is no single method that is capable of assessing this complicated matrix of sesame oil properties. As a result of low production yields and high processing costs, pressed sesame oil is more expensive than other edible oils and a frequent target of adulteration with cheaper refined oils, such as peanut oil, soybean oil, canola oil, and corn oil [4]. Additionally, sesame flavor additives have been also added to refined vegetable oils, which have been subsequently marketed as sesame oil. The food industry needs a quick and sensitive method to characterize pressed sesame oils and to detect adulterated sesame oil. Most methods, such as HPLC, GC-MS, NIR/MIR and Raman spectroscopy, for characterizing edible oil are based on differences in chemical composition [5,6,7,8,9,10]. Electronic noses (e-nose) have been developed to mimic human olfaction and used to assess food quality [11]. In an e-nose, sensor arrays are employed to record smell and flavor (volatiles) response signals as a global fingerprint. The e-nose technology is a rapid method for discriminating materials (foods) with different smells.

However, the signals of e-nose generally have no direct correspondence to any individual chemical components, which differentiates an e-nose from such traditional analytical equipment as gas chromatography. Pattern recognition methods are essentially required to analyze the global fingerprint generated by the sensor array in the e-nose. Simon reported that many data analysis techniques had been used in processing e-nose data [12]. Unsupervised methods, such as principal component analysis (PCA), and clustering algorithms, were used as exploratory techniques, while supervised methods, such as principal component regression (PCR), linear discriminant analysis (LDA), k-nearest neighbour algorithm (KNN), partial least squares (PLS) regression, soft independent modelling class analogy (SIMCA), and neural networks (NN), were used as classification techniques [12]. Support vector machine (SVM) also was used as a prediction technique [13,14,15]. Gromski had applied four types of classifier in analyzing e-nose data and found that LDA and SVM with a polynomial kernel resulted in the best overall results [16]. A wide variety of pattern recognition methods can be used in interpreting e-nose data, and the question that is often asked is: which one was the most suitable? Selecting a suitable method for analyzing a specific type of e-nose data is still a difficult task.

No Free Lunch Theorems (NFL) indicates that no single method is optimal for solving all problems [17,18]. Each set of data therefore may require the choice of an optimal (or a near-optimal) algorithm for that particular dataset. Some studies have been reported in the literature focusing on how to choose a good method for detecting oil adulteration. For example, Concepción found LDA method was satisfactory when used to analyze e-nose data for detecting virgin olive oil adulterated with other oils, with an overall accuracy above 95% [19]. Zhang also used an e-nose with LDA to discriminate refined sesame and camellia oils mixed with refined maize oil, and achieved an accuracy of 94.5% [20].

The objective of this study was to evaluate different methods for interpreting e-nose data in characterizing sesame oils and their blends (adulteration). The methods evaluated in this study represented the majority of the classification methods reported in the literature for e-nose analysis.

## 2. Experimental Section

### 2.1. Sample Preparation

Four edible oil samples from different sources were used to generate e-nose data, including three sesame oil samples (C, H, and R), one refined oil produced in India, one cold-pressed and one hot-pressed oil produced in China; and one soybean oil sample (S) produced in Argentina (Table 1). The three sesame oil samples were obtained from a plant in Anhui, China; and the soybean oil sample was purchased at a local supermarket in Nanjing, Jiangsu, China

**Table 1:** Oil sample information.

No.	Abbr.	Name	Processing	Origin
1	S	Soybean oil	Refined	Argentina
2	R	Sesame oil	Refined	India
3	C	Sesame oil	Cold-pressed	Anhui, China
4	H	Sesame oil	Hot-pressed	Anhui, China

Adulterated sesame oil samples (blends) were prepared by mixing pressed sesame oils with refined soybean or sesame oil at different ratios in four groups (Table 2). These ratios were chosen to simulate various flavor oil blends in markets. The adulterated samples were mixed by pipetting oils a 20 mL sealed plastic tube. After being vigorously shaken in a shaker for 30 min, the samples were allowed to equilibrate overnight at room temperature prior to analyses. There were eight replicated samples for each adulteration ratio.

**Table 2:** Adulterated sesame oil samples.

NAME	Mixing A with B (A+B)	Adulteration Level (V_B_/V_A+B_)
HS	Hot-pressed sesame oil + Refined Soybean oil	0%, 1%, 5%, 10%, 20%, 30%, 40%, 50%, 60%, 70%, 80%, 90%, 100%
CS	Cold-pressed sesame oil + Refined Soybean oil	0%, 1%, 5%, 10%, 20%, 30%, 40%, 50%, 60%, 70%, 80%, 90%, 100%
HR	Hot-pressed sesame oil + Refined sesame oil	0%, 1%, 5%, 10%, 20%, 30%, 40%, 50%, 60%, 70%, 80%, 90%, 100%
CR	Cold-pressed sesame oil + Refined sesame oil	0%, 1%, 5%, 10%, 20%, 30%, 40%, 50%, 60%, 70%, 80%, 90%, 100%

### 2.2. E-Nose Measurement and Feature Selection

An Alpha MOS FOX-3000 e-nose (Alpha MOS, Toulouse, France) equipped with an auto-sampler (HS 100, CTC Analytics, Nantes, France) was used to collect data. The e-nose had 12 metal oxide semiconductor (MOS) sensors. Based on sensor coating materials, the LY sensors (LY2/G, LY2/AA, LY2/Gh, LY2/gCT1, LY2/gCT, and LY2/LG) were p-type semiconductors, while the P and T sensors (T30/1, P10/1, P10/2, P40/1, T70/2, and PA2) were n-type semiconductors.

**Figure 1:**
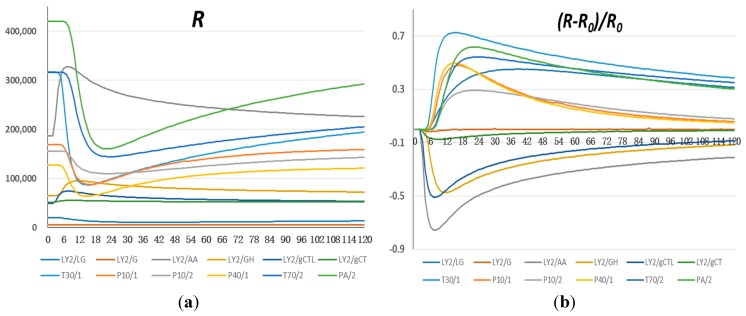
Sensor responses to refined oil. (**a**) Original sensor signals; (**b**) Sensor resistance ratios.

Oil samples (1 mL) were transferred to screw-capped 10-mL bottles and placed in the auto sampler. The samples were heated at 60 °C and shaken at 500 rpm for 300 s to generate sufficient volatiles in the headspace. An aliquot (1000 μL) was taken from the headspace of the bottles, injected into the sensor chambers of the e-nose, and flushed over the sensors at 150 mL/min. Upon injecting the sample, data were recorded every second over 120 s (Figure 1a). The default time period in-between samples injections was 30 min, but 15 min was used in this study, which was sufficient for the sensor signals to return to the baseline.

As sample gases flowed over the sensors, sensors’ resistance (R) changed. Figure 1a shows typical signal patterns of sensors for refined oil. It can be seen that each sensor responded differently, *i.e.*, the resistance of some sensors increased, some decreased, and some barely changed. Therefore, a ratio (R−R_0_)/R_0_ was commonly used to reflect the relative changes in sensor resistance (Figure 1b), where R_0_ is the sensor’s resistance baseline and R is the real-time resistance. Also, the maximum resistance ratio (MRR) was considered as the each sensor’s feature for one measurement.

### 2.3. Data Analysis Methods and Implementation

The LDA, PCA and PLS are fundamental methods used in chemometrics [12]. The PLS-DA is an application of regression for the optimal separation of classes using a series of dummy variables as Y variables to represent each group [21]. LASSO is a linear regression method which minimizes the usual sum of squared errors, with a bound on the sum of the absolute values of the coefficients [22]. The above methods of analysis, as well as ANOVA, were performed with SAS/STAT (SAS^®^9.4, SAS Institute, Cary, NC, USA). A SAS macro was developed to graphically illustrate the results of analysis, with confidence ellipses containing 95% of each sample’s MRR [23].

The R 3.2.2 software environment (http://cran.r-project.org/) was used for the following data analysis. The SVM has been widely used for pattern recognition [24]. SVM analysis was implemented using e1071 [25]. Four types of kernels were used in SVM models: linear, polynomial, radial, and sigmoid. A grid search procedure was performed on the training dataset to find the optimal cost and gamma parameters. The KNN is a non-parametric method used for classification and regression. The distance criterion used in the present KNN analysis was the Euclidean distance, which was implemented using FNN [26]. The RF analysis relies on constructing a series of tree-based “learners” which use a subset of the input space. The method can also be used for variable selection, or to understand the important features (variables) that are used for prediction, which was implemented using randomForest [27]. The optimization of the most appropriate parameters for SVM and RF was calculated by using the built-in grid search function. The following R-packages were employed for SVM, KNN and RF analyses, respectively:

(1) “Misc Functions of the Department of Statistics, Probability Theory Group (Formerly: E1071), TU Wien” (Version 1.64) was used for SVM [25].
(2) Fast Nearest Neighbor Search Algorithms and Applications” (Version 1.1) was used for KNN classification and regression [26].
(3) “Breiman and Cutler’s random forests for classification and regression” (Version 4.6–10) was used for random forests [27].

### 2.4. Data Preparation and Model Validation

Based on the experimental design, a total of 104 observations (13 classes and eight repetitions) were to be collected for each of the four test groups. We actually collected 99, 100, 104 and 104 observations for HS, CS, HR and CR, respectively, and some samples were in the HS and CS groups were not measured properly by the e-nose because of auto-sampler malfunction. Each observation contains 12 MRR values and one label (the oil type or adulteration level).

The performance of a model relates to its prediction capability on independent test data. In order to compare classification and prediction performance of different methods, it is essential to use cross validation. The hold-out method is the simplest kind of cross validation, in which the data set is divided into two independent sets, called the training set and the testing set. Typically, the function approximator fits a function using the training set only. However, the performance evaluation based on the hold-out method can have a high variance and the evaluation may be significantly different depending on how the data division is made. The k-fold cross validation is a common way to improve the reliability over the hold-out method. In the k-fold method, the data set is divided into k subsets, and the hold-out method is then repeated k times. Each time, one of the k subsets is used as the test set and the other k − 1 subsets are put together to form a training set. Then, the average error across all k trials is computed. In this study, the k-fold cross validation with k = 10 was used to generate multiple training and test data sets. We applied 1000 runs to determine the estimation accuracy, AUC, RMSEC, and RMSEP. The 100 runs and 10,000 runs were also tested, but we found that the results were not much different from 1000 runs.

### 2.5. Accuracy and MAUC

Accuracy, defined as the percentage of predictions that are correct, was used as a performance measure of classification methods. However, accuracy alone might not give an overall picture of classification performance. Sensitivity, specificity and ROC plots could also be used to evaluate the method performance. However, for multi-classification in this paper, an excessively large table would be required to accommodate sensitivity and specificity or number of ROC plots, which was considered not to be feasible for this paper. Instead, the concept of AUC (Area under ROC curve), which is often used as a measure of quality of classification models, was adopted in this study to supplement the accuracy measurement to assess the performance of classification methods. The AUC value was calculated as follows [28].

(1) $$AUC=1-\frac{\frac{x}{n}+\frac{k-x}{m}}{2}$$

where *m*, *n*, *k* and *x* represent the number of positive examples, negative examples, classification errors and false positive examples, respectively. 

We extended the AUC to multi-classification (MAUC) by treating a multiclass classification problem as a set of binary classification. Let *c* be the number of classes, and MAUC was calculated as the mean all class’ AUC:

(2) $$MAUC=\;\overset{c}{\underset{i=1}{\sum }}AUC\left(i\right)/c\;$$

## 3. Results and Discussion

### 3.1. Comparison of Sensor Signals among Four Groups of Oil Samples

It was observed that all 12 sensors were responsive to all oil samples in Table 3. Sensors’ MRRs for hot- and cold-pressed oil samples (C and H) were in the range of −0.831–0.753, −0.762–0.742, respectively, and those for refined sesame and soybean oil samples (R and S) were in the range of −0.182–0.409, −0.040–0.348, respectively. For 10 of the 12 sensors, the sorted order by MRR was: C > H > R > S. This indicated: (i) signals from cold-pressed sesame oil samples were generally stronger than those from hot-pressed oil samples; and (ii) pressed oil samples (C and H) generated stronger signals than refined oil (R); and (iii) all sesame oil sample (C, H and R) had stronger signals than did the soybean oil sample (S). Statistical analysis indicated the MRRs for cold-pressed sesame oil samples were significantly higher than those for hot-pressed oil samples for all sensors except LY2/LG and T30/1 (*p* < 0.05). This seemed to be contradictory to the general observation that hot-pressed oils are always more “pungent” to human noses than cold-pressed oils because of such chemical reactions as Maillard reaction occurring during high temperature processing [29]. The reason for the “abnormal” observation in this study was probably due to the procedure of sampling volatiles from the vials. The oil samples in vials were incubated at 60 °C for 300 s prior to taking headspace gas (volatile) for injection into the e-nose. This incubation temperature probably had more effect on the cold-pressed oil (*i.e.*, causing more volatiles to be released) than did on hot-pressed oil.

**Table 3:** ANOVA of e-nose maximum resistance ratio (MRR) values for four oil samples.

Sensor	LY2/LG	LY2/G	LY2/AA	LY2/GH	LY2/gCTL	LY2/gCT	T30/1	P10/1	P10/2	P40/1	T70/2	PA/2
C *	0.43 ^B^ **	−0.44 ^A^	−0.80 ^D^	−0.53 ^D^	−0.60 ^C^	−0.09 ^D^	0.74 ^A^	0.55 ^A^	0.37 ^A^	0.56 ^A^	0.57 ^A^	0.66 ^A^
H	0.47 ^A^	−0.06 ^B^	−0.74 ^C^	−0.48 ^C^	−0.51 ^B^	−0.08 ^C^	0.73 ^A^	0.51 ^B^	0.32 ^B^	0.52 ^B^	0.54 ^B^	0.64 ^B^
R	0.05 ^C^	−0.01 ^B^	−0.17 ^B^	−0.08 ^B^	−0.020 ^A^	−0.02 ^B^	0.31 ^B^	0.38 ^C^	0.27 ^C^	0.40 ^C^	0.17 ^C^	0.22 ^C^
S	0.03 ^D^	−0.01 ^B^	−0.04 ^A^	−0.02 ^A^	−0.020 ^A^	−0.01 ^A^	0.15 ^C^	0.32 ^D^	0.24 ^D^	0.34 ^D^	0.09 ^D^	0.12 ^D^

* C: Cold-pressed sesame oil; H: Hot-pressed sesame oil; R: Refined sesame oil; S: refined soybean oil; ** The same letter in the same column indicates no significant difference at *p* < 0.05.

For each of the 12 sensors, the absolute MRR for pressed oil samples (C or H) was significantly higher than those for refined oil samples (S and R) (*p* < 0.05). This could be attributed to the fact that refined oil had gone through a series of operations such as decolorization and deodorization, during which some volatiles were removed. In contrast, pressed sesame oil was only physically pressed, and thus it retained most volatiles. In other words, pressed oil samples had more volatiles in the headspace of sampling vials than did the refined oil samples, causing stronger responses of e-nose sensors. Similar observations were made by other researchers. For example, in studying the volatile profiles of several oils using gas chromatography, Zhong *et al.* observed that refined oils had a lower total peak area than the corresponding crude or cold-pressed oil in the chromatogram [30].

It was also observed that there were differences in MRRs between refined sesame oil and refined soybean oil. This deference reflected different volatiles contained in the two oils, and this difference was still measurable even after refining, as a human nose could normally do.

**Figure 2:**
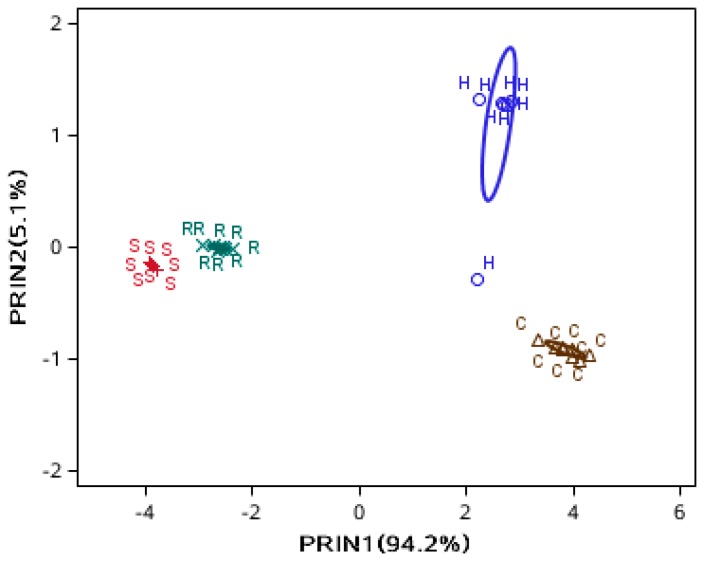
PC1 and PC2 score plot from PCA analysis for four oil samples. H: Hot-pressed sesame oil; C: Cold-pressed sesame oil; R: Refined sesame oil; S: Refined soybean oil.

To obtain an overview of the variation in the e-nose signals, PCA was performed to reduce data dimensions for observing the trends, grouping data, and detecting outliers. The PCA diagram was firstly used to reveal any possible groupings for oil samples without any adulteration. The separation between the four oil samples was very clear from Figure 2. The two pressed oils were far from each other while two refined oils were close, indicating that the flavor of hot-pressed sesame oil was very different from that of cold-pressed sesame oil and the flavor of refined soybean oil was similar to that of refined sesame oil.

### 3.2. Data Dimensionality Reduction and Visualization of Adulteration Level Separation

**Figure 3:**
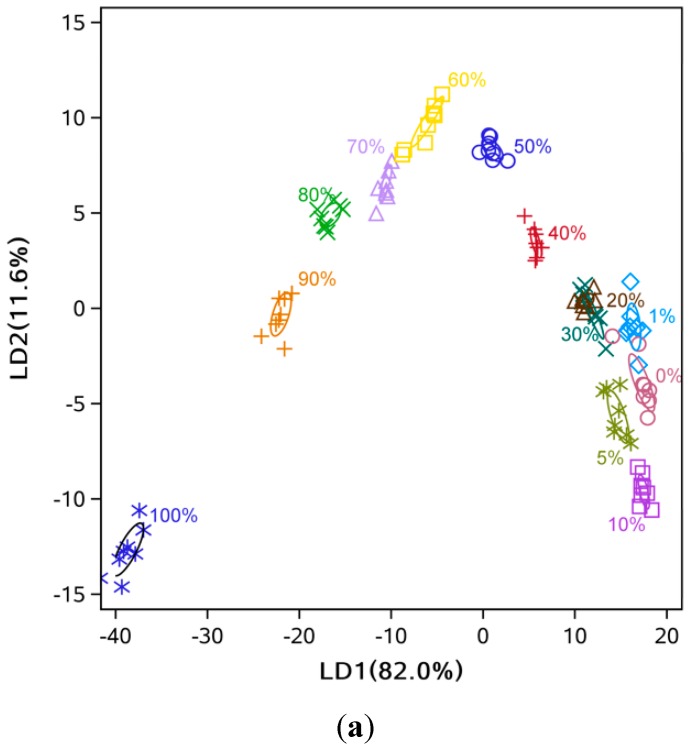

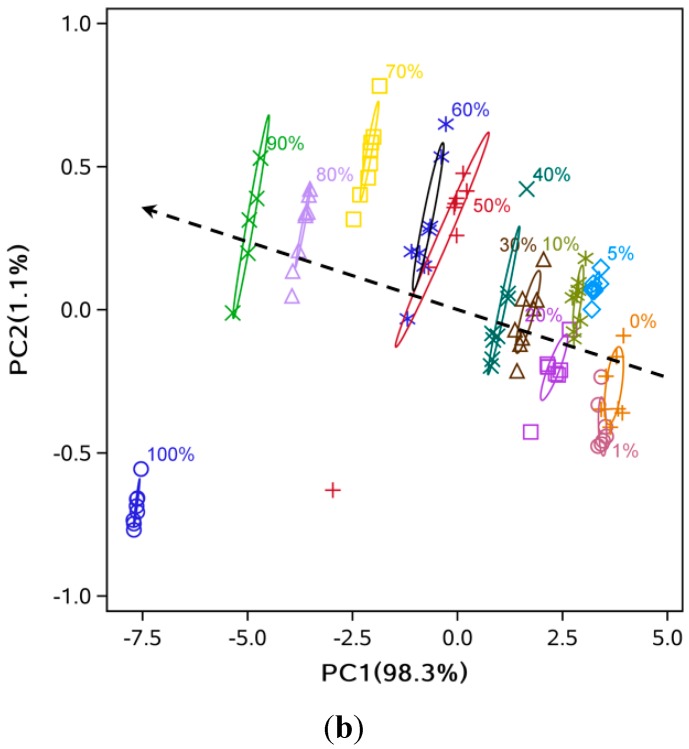
PC1 and PC2 score plot from PCA analysis for sesame oil mixed with soybean oil. (**a**) hot-pressed oil mixed with refined soybean oil; (**b**) cold-pressed oil mixed with refined soybean oil.

**Figure 4:**
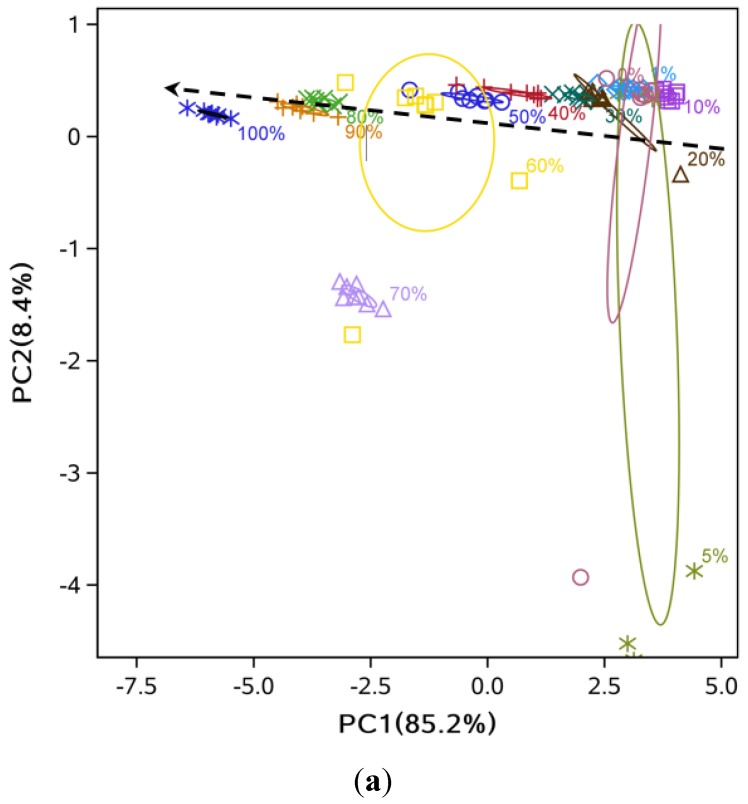

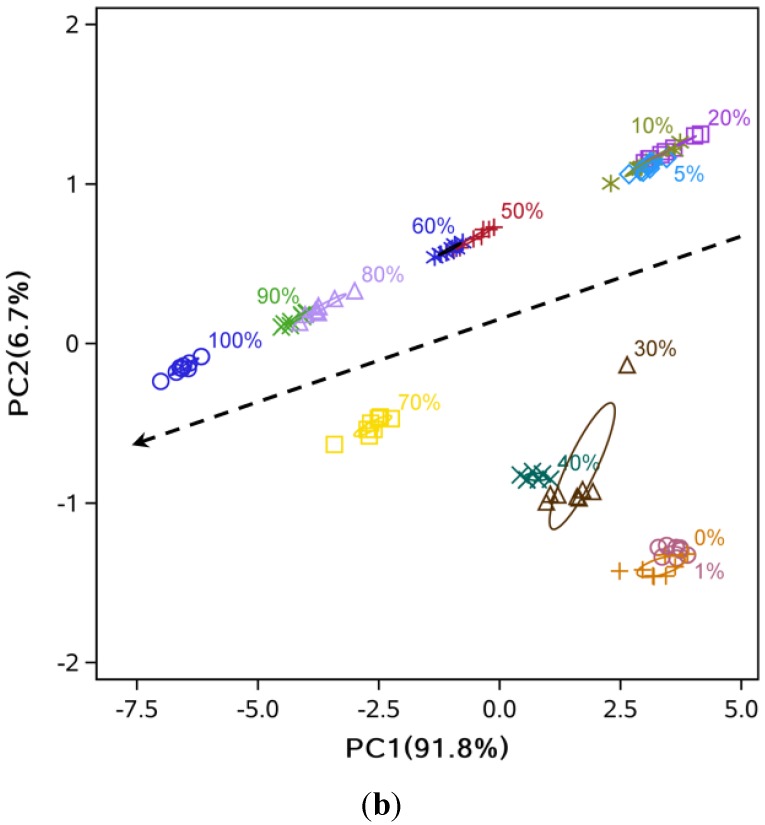
PC1 and PC2 score plot from PCA analysis for mixtures of different sesame oils. (**a**) Hot-pressed sesame oil mixed with refined sesame oil; (**b**) Cold-pressed sesame oil mixed with refined sesame oil.

For the hot-pressed sesame oil with different adulteration levels of soybean oil, the separation between the eight classes was very clear, with slight overlapping among 0%, 1% and 5% adulteration levels (Figure 3a). A similar observation was made for the hot-pressed sesame oil mixed with soybean oil with overlapping between 0% and 1% samples (Figure 3b). It could be concluded that the e-nose was capable of distinguishing different levels of adulteration but with lower accuracy at very low (<5%) levels of adulteration (Figure 3 and Figure 4). Also, some data points located far from their ellipse center were considered as outliers by visual inspection. Specifically, there were one point each at 30% and 80% adulteration for hot-pressed oil (Figure 3a), and one point each at 40% and 50% adulteration for cold-pressed oil ( Figure 3b).

For cold- and hot-pressed oil samples mixed with refined sesame oil, the separation at high levels of adulteration (≥20%) was clear, but heavy overlapping at low adulteration levels was observed (Figure 4). This indicated that pressed sesame oil and refined sesame oil had smaller differences compared to refined soybean oil, as expected.

**Figure 5:**
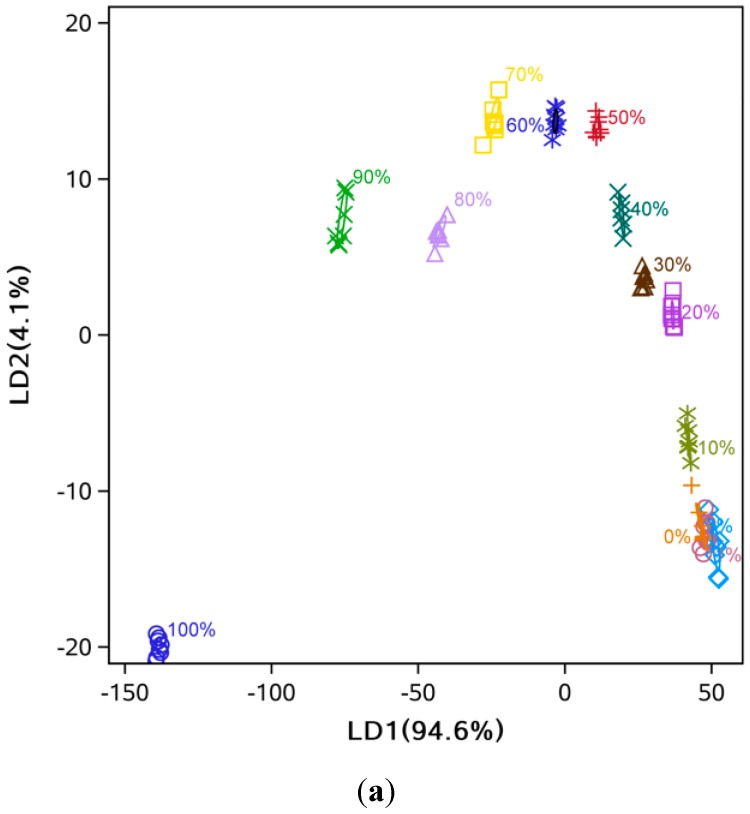

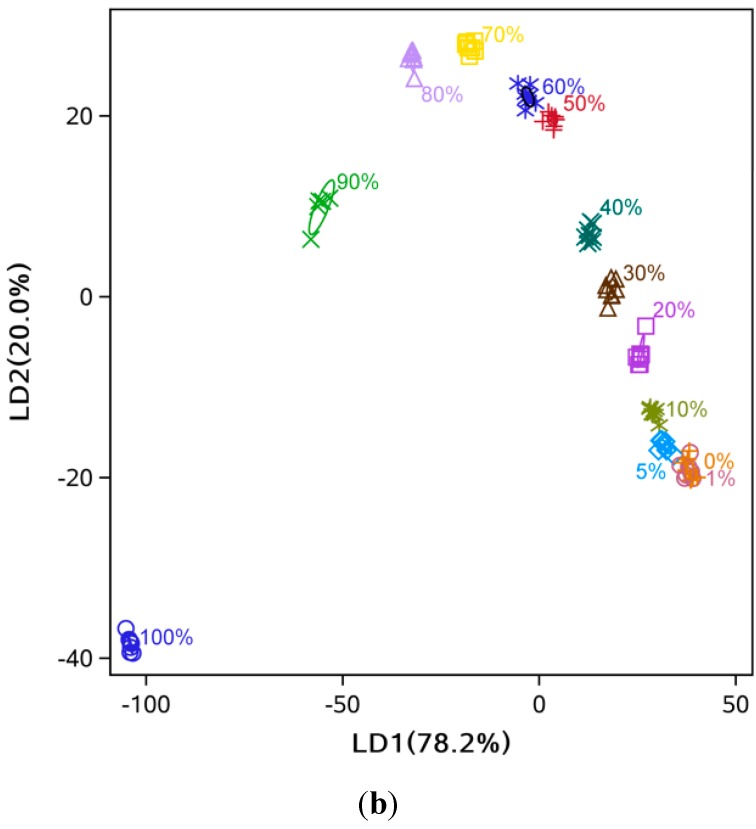
LD1 and LD2 score plot from LDA analysis. (**a**) Hot-pressed sesame oil mixed with refined soybean oil; (**b**) Cold-pressed sesame oil mixed with refined soybean oil.

**Figure 6:**
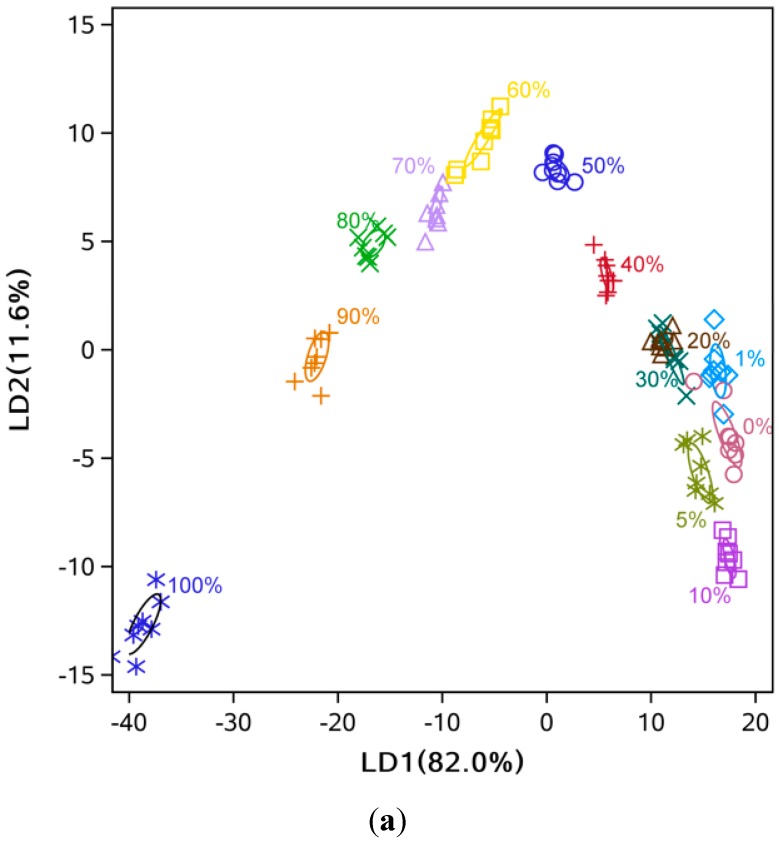

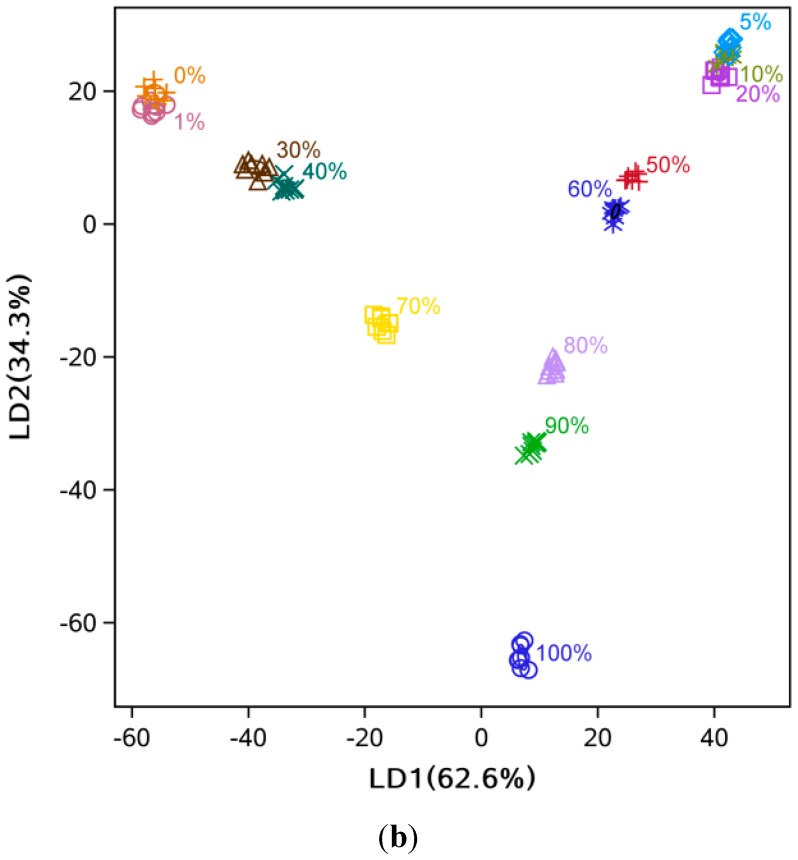
LD1 and LD2 score plot from LDA analysis. (**a**) Hot-pressed sesame oil mixed with refined sesame oil; (**b**) Cold-pressed sesame oil mixed with refined sesame oil.

The LDA showed that high discriminant accuracy could be achieved with only two LDs (Figure 5 and Figure 6). For example, the discriminant accuracy was almost 100% for cold-pressed sesame oil mixed with refined soybean oil (Figure 5b). Some overlapping was still observed at low levels of adulteration (<10%) (Figure 5a and Figure 6b). Additionally, the samples’ points follow a zigzag along LD1 and LD2 because of the two-dimensional diagram’s limitation in showing the separation between groups, and distance of some points could be easily misunderstood. For example, the 0% and 30% samples are closer than the 0% and 5% samples. Similar to the PCA analysis, comparing Figure 5 with Figure 6 indicated that mixtures of pressed sesame oil with refined soybean oil separated more easily than mixtures of pressed and refined sesame oils.

### 3.3. Comparison of Classification Methods

For all five methods applied in this study, the overall accuracies were between 25.0% and 96.3% (Table 4). The classification accuracy of KNN method ranged between 95.0% and 96.3%, with the highest at k = 1. The accuracy of LDA was comparable to KNN at 96.2%. For a given dataset, the performance of SVM depended on the selection of kernel, kernel parameters, and soft margin parameter C. A sigmoid kernel resulted in a very low accuracy of 25.0%, while a linear kernel achieved the best average accuracy of 95.1%. The best combination of polynomial kernel parameters was selected by a grid search, but the accuracy for HR was only 68.2%. The PLS-DA and RF both performed poorly and had classification accuracies below 90%. SVM, RF and KNN are commonly accepted as high performance methods for industrial and commercial data [31]. However, not all of them worked well in this study.

Based on the accuracy shown in Table 4, the five calcification methods were ranked as follows: KNN (k = 1), LDA, SVM (linear kernel), RF, and PLS-DA. The difference between KNN (k = 1) and LDA was negligible (96.3% *vs.* 96.2%). MAUC values for each group were consistent with accuracy (Table 4). In other words, the ranking of the five methods by their MAUC values was the same as that by accuracy. It should be noted that accuracy was determined based on a single classification threshold, while MAUC summarized the performance based on all thresholds, and was therefore much more informative, especially for highly skewed datasets [32].

For all five methods, the accuracy and MAUC for HS and CS was higher than that for HR and CR (Table 4). Taking the KNN (k = 1) as an example, the accuracy was above 98% for HS and CS, and was about 94% for the HR and CR.

**Table 4:** Comparison of accuracy and MAUC among five classification methods, based on 1000 runs of k-fold (k = 10) cross-validation.

METHOD		Accuracy (MEAN ± SD) MAUC (95% CI)				Overall Accuracy/Overall MAUC
		HS	CS	HR	CR	
LDA		97.0% ± 5.3% 0.982~0.986	98.9% ± 3.2% 0.993~0.995	94.3% ± 6.7% 0.967~0.971	94.7% ± 6.6% 0.969~0.974	96.2%/0.980
PLS-DA		68.4% ± 16.0% 0.835~0.845	56.1% ± 15.2% 0.776~0.785	56.5% ± 18.4% 0.778~0.790	50.0% ± 22.4% 0.748~0.762	57.8%/0.794
KNN	K = 1	98.3% ± 4.3% 0.990~0.993	98.9% ± 3.2% 0.993~0.995	93.9% ± 7.0% 0.966~0.970	93.7% ± 7.2% 0.965~0.970	96.3%/0.981
	K = 2	97.4% ± 5.0% 0.984~0.988	98.2% ± 4.2% 0.988~0.991	94.0% ± 7.1% 0.964~0.969	94.2% ± 7.2% 0.964~0.969	96.0%/0.977
	K = 3	97.1% ± 5.6% 0.984~0.988	98.7% ± 3.4% 0.993~0.994	92.4% ± 7.5% 0.957~0.962	93.2% ± 7.4% 0.963~0.968	95.4%/0.977
	K = 4	96.1% ± 6.0% 0.977~0.981	98.2% ± 4.1% 0.989~0.992	93.2% ± 7.3% 0.960~0.966	94.3% ± 7.1% 0.966~0.971	95.5%/0.975
	K = 5	95.7% ± 6.6% 0.976~0.980	98.6% ± 3.6% 0.991~0.994	93.2% ± 7.6% 0.961~0.967	93.2% ± 8.0% 0.962~0.967	95.2%/0.975
	K = 6	95.1% ± 6.7% 0.971~0.976	98.3% ± 4.0% 0.989~0.992	93.2% ± 7.5% 0.961~0.967	93.2% ± 7.9% 0.961~0.967	95.0%/0.973
SVM	linear	94.1% ± 7.8% 0.966~0.970	97.8% ± 4.7% 0.986~0.990	91.4% ± 8.6% 0.952~0.958	97.0% ± 5.0% 0.982~0.986	95.1%/0.974
	polynomial	93.6% ± 8.3% 0.964~0.969	97.1% ± 5.3% 0.983~0.987	68.2% ± 13.5% 0.832~0.841	97.9% ± 4.4% 0.987~0.990	89.2%/0.944
	RBF	92.6% ± 8.6% 0.959~0.965	95.8% ± 6.2% 0.976~0.980	89.1% ± 9.9% 0.940~0.947	91.0% ± 9.4% 0.952~0.957	92.1%/0.960
	sigmoid	34.7% ± 15.0% 0.662~0.671	8.1% ± 12.8% 0.497~0.506	42.8% ± 16.0% 0.693~0.703	14.3% ± 11.8% 0.568~0.577	25.0%/0.610
RF		91.44% ± 9.3% 0.953~0.959	93.8% ± 7.2% 0.966~0.970	84.1% ± 11.1% 0.913~0.920	83.3% ± 11.7% 0.909~0.917	88.1%/0.939

Our findings were in general agreement with what has been reported in the literature concerning the LDA and SVM methods [16,19,20]. The KNN performed the best in this study, but little information could be found in the literature on the use of KNN for analyzing e-nose data to compare with. Comparison with other studies [6] revealed that the three top ranked methods, namely KNN, LDA, and SVM, which all had the overall accuracy greater than 95% in analyzing the e-nose data, were comparable or better than other commonly used methods. For example, GC-MS was used to detect the adulteration of soybean oil with other oils and the accuracy was 92% [6].

### 3.4. Prediction of Adulteration Level

Five models, namely LASSO [22], PLS [21], SVM [24,25], KNN [26] and RF [27], were used to predict the adulteration levels in sesame oil blends, with the MRRs of the 12 e-nose sensors as input (independent variable). Every group of dataset was divided into two parts (9:1) 1000 times randomly, and the root mean square of error (RMSE) was calculated (Table 5). Optimization of models’ parameters and cross validation were performed to avoid overfitting, so that the differences between all pairs of RMSEC and RMSEP were minimal. All models performed well in most cases, except SVM with sigmoid kernel. KNN with k = 1 or 2 had the lowest value of RMSEP for HS, HR and CR, while LASSO, PLS and KNN (k = 1) had the lowest RMSEP for CS. RF performed better in than LASSO and PLS for HR and CR, but not for HS and CS. On the whole, KNN with k = 1 or 2 performed best in predicting the adulteration levels in all groups, with *R*^2^ of 0.99, while the performance of LASSO, PLS, SVM with linear or RBF kernel, and RF was acceptable.

**Table 5:** Comparison of prediction performance of five methods, based on 1000 runs of k-fold (k = 10) cross-validation.

METHOD		RMSEC/RMSEP (Average Value)			
		HS	CS	HR	CR
LASSO		2.01/2.15	1.79/1.92	5.53/6.06	4.64/4.69
PLS		1.70/1.95	1.34/1.55	4.04/4.54	4.37/5.63
KNN	K = 1	1.74/1.12	2.79/1.79	3.47/2.65	1.41/0.80
	K = 2	1.71/1.25	3.08/2.40	3.26/2.63	1.82/1.42
	K = 3	1.82/1.38	3.28/2.53	3.67/3.12	2.30/2.05
	K = 4	2.02/1.61	3.37/2.61	3.97/3.41	2.56/2.37
	K = 5	2.22/1.86	3.51/2.83	4.15/3.60	2.76/2.66
	K = 6	2.53/2.22	3.71/3.10	4.30/3.78	2.94/2.86
SVM	linear	2.06/2.39	1.99/2.18	3.85/4.52	3.17/5.00
	polynomial	8.96/10.22	2.05/2.33	2.19/3.43	2.18/3.54
	RBF	2.19/2.43	2.13/2.66	2.37/3.24	2.34/3.36
	sigmoid	6165.29/5944.58	7699.49/7517.63	1477.14/1506.62	1923.44/1932.49
RF		2.55/2.18	3.02/2.38	4.51/3.79	3.55/3.39

## 4. Conclusions

All 12 metal oxide semiconductor (MOS) sensors in the Alpha MOS FOX-3000 electronic nose were responsive to sesame oils, but sesame oils processed with different methods resulted in different levels of sensor response. Specifically, cold-pressed sesame oil resulted in the strongest response, followed by hot-pressed oil, and then refined oil. The responses of e-nose sensors to all three types of sesame oil were stronger than that to refined soybean oil, which was less aromatic compared to sesame oils.

Among the five discrimination methods for analyzing e-nose data collected on sesame oil blends with sesame oil contents, the performance of LDA, KNN, and SVM was clearly better than PLS-DA and RF. The three top ranked methods (LDA, KNN, and SVM) achieved an overall classification accuracy above 95% and MAUC above 0.97. This performance was comparable to or better than some high-end instruments such as Fourier-transformed infrared spectroscopy. In terms of predicting the adulteration level in sesame oil blends, KNN with small k (k = 1 or 2) performed best, while the performance of LASSO, PLS, SVM (with linear or RBF kernel) and RF was adequate.

It is clear that the e-nose coupled with appropriate classification and prediction methods was capable of detecting adulterated pressed sesame oil. It should be noted that blends of pressed and refined sesame oils were more difficult to be distinguished than were blends of pressed sesame oil and refined soybean oil.
